# Human Mesenchymal Stem Cells Protect Human Islets from Pro-Inflammatory Cytokines

**DOI:** 10.1371/journal.pone.0038189

**Published:** 2012-05-30

**Authors:** Telford Y. Yeung, Karen L. Seeberger, Tatsuya Kin, Adetola Adesida, Nadr Jomha, A. M. James Shapiro, Gregory S. Korbutt

**Affiliations:** 1 Department of Surgery, 5-002 Li Ka Shing Centre for Health Research Innovation, University of Alberta, Edmonton, Alberta, Canada; 2 Alberta Diabetes Institute, 5-002 Li Ka Shing Centre for Health Research Innovation, University of Alberta, Edmonton, Alberta, Canada; University of Bremen, Germany

## Abstract

Transplantation of human islets is an attractive alternative to daily insulin injections for patients with type 1 diabetes. However, the majority of islet recipients lose graft function within five years. Inflammation is a primary contributor to graft loss, and inhibiting pro-inflammatory cytokine activity can reverse inflammation mediated dysfunction of islet grafts. As mesenchymal stem cells (MSCs) possess numerous immunoregulatory properties, we hypothesized that MSCs could protect human islets from pro-inflammatory cytokines. Five hundred human islets were co-cultured with 0.5 or 1.0×10^6^ human MSCs derived from bone marrow or pancreas for 24 hours followed by 48 hour exposure to interferon-γ, tumor necrosis factor-α and interleukin 1β. Controls include islets cultured alone (± cytokines) and with human dermal fibroblasts (± cytokines). For all conditions, glucose stimulated insulin secretion (GSIS), total islet cellular insulin content, islet β cell apoptosis, and potential cytoprotective factors secreted in the culture media were determined. Cytokine exposure disrupted human islet GSIS based on stimulation index and percentage insulin secretion. Conversely, culture with 1.0×10^6^ bMSCs preserved GSIS from cytokine treated islets. Protective effects were not observed with fibroblasts, indicating that preservation of human islet GSIS after exposure to pro-inflammatory cytokines is MSC dependent. Islet β cell apoptosis was observed in the presence of cytokines; however, culture of bMSCs with islets prevented β cell apoptosis after cytokine treatment. Hepatocyte growth factor (HGF) as well as matrix metalloproteinases 2 and 9 were also identified as putative secreted cytoprotective factors; however, other secreted factors likely play a role in protection. This study, therefore, demonstrates that MSCs may be beneficial for islet engraftment by promoting cell survival and reduced inflammation.

## Introduction

Islet transplantation is an attractive alternative treatment to daily insulin injections for patients with type 1 diabetes [1]. Following the Edmonton Protocol, nearly 90% of islet transplant recipients remained insulin independent at one year [2]; however, only 10% of the recipients were insulin independent at five years’ post transplant [3]. This loss of graft function may be attributed to various factors including toxicity of immunosuppressive drugs [3], immune rejection [3], and inadequate supply of islet precursor cells for β cell replacement [4]. However, immediately after transplantation, inflammation plays a significant role in the loss of islet function [5], [6]. This inflammatory response is characterized by elevated interferon-γ (IFN- γ), tumor necrosis factor-α, (TNF-α) and interleukin 1β (IL-1β) [5], [7]. Rodent and human islet cells exposed to these pro-inflammatory cytokines lose glucose responsiveness and express increased markers of apoptosis [8], [9]. In addition to β-cell cytotoxicity, these cytokines exacerbate the inflammatory response by recruiting and activating immune cells such as macrophages [5], [10]. Decreasing cytokine expression, inhibiting cytokine activity or inhibiting macrophage activity improves the function of transplanted islets [5], [7], [10]. Thus, minimizing inflammation at the transplant site during engraftment may help to prolong islet graft function and maintain long-term insulin independence.

Control of islet graft inflammation may be achieved by co-transplantation of islets with multipotent mesenchymal stromal cells, also known as mesenchymal stem cells (MSCs) [11]. These stromal cells are connective tissue derived stem cells with immunomodulatory and regenerative properties [12], [13]. They also secrete anti-inflammatory proteins and suppress the activity of various immune cells such as alloantigen activated T and B lymphocytes [12], [13]. Moreover, MSCs can secrete growth factors that improve tissue survival, stimulate angiogenesis and facilitate tissue engraftment in animal models of myocardial infarction, diabetes and graft versus host disease [12], [13].

In cell culture, the trophic effects of MSCs have also been reported with rodent and human islets [14]–[18]. However, the capacity of human MSCs to protect human islets from pro-inflammatory cytokines has not been studied. Therefore, in this study, we examined the cytoprotective effect of bone marrow and pancreatic derived [19] MSCs on human islets in vitro. We co-cultured human islets with human MSCs and measured islet cell survival and function after exposure to a pro-inflammatory cytokine cocktail. We report that MSC aggregates preserve glucose stimulated insulin secretion (GSIS), prevent islet β cell apoptosis, and identify possible secreted MSC factors that mediate this protection. Thus, co-administering MSCs may be beneficial in prolonging islet graft survival.

## Materials and Methods

### Ethics Statement

Human pancreases were procured from cadaveric donors after written informed research consent was provided by donor relatives. Written ethical approval for this research study was provided by the University of Alberta’s Health Research Ethics Board – Biomedical panel (study ID: Pro00001416).

### Preparation of Human Islets

Human pancreases (ages ranging from 16 to 71, n = 23) were processed according to islet isolation protocols previously described by our group [1], [4]. Islet enriched fractions (10–30% dithizone positive) were cultured in Roswell Park Memorial Institute (RPMI) 1640 (Gibco, Carlsbad CA) medium supplemented with 0.5% w/v fraction V bovine serum albumin (BSA, Sigma-Aldrich, Oakville, Canada) and 1.0% v/v insulin-transferrin-selenium (ITS, Sigma-Aldrich).

### Preparation of Human Bone Marrow and Pancreatic Derived MSCs

To prepare bone marrow derived MSCs (bMSCs), human bone marrow was extracted from six patients aged 24, 42, 45, 46, 62, and 69 years (Division of Orthopedic Surgery, University of Alberta) following informed consent [20]. For expansion, cells were plated in Modified Essential Medium alpha (MEMα, Cellgro Manassas, VA) supplemented with 2.5 ng/mL basic fibroblast growth factor (bFGF, Millipore, Billerica, MA), 10% fetal bovine serum (FBS, Gibco), 1 mM sodium pyruvate (Gibco), 10 mM HEPES (Gibco), 100 U penicillin/1000 U streptomycin (Biowhittaker, Walkersville, MD) at a density of 166,000 cells per cm^2^. Non-adherent cells were removed by changing the medium every 2–3 days. Once conﬂuent, the cell monolayer was washed with versene and was detached with 0.05% v/v trypsin-EDTA (Invitrogen, Carlsbad CA). Cells were counted and re-seeded into supplemented MEMα culture medium at a density of 5000–10000 cells/cm^2^.

To prepare human pancreatic derived MSCs (pMSCs), human pancreatic tissue from islet depleted fractions were obtained from seven donors aged 36, 44, 49, 49, 52, 57, and 64 years and were cultured in RPMI 1640 with 0.5% w/v BSA and 1.0% v/v ITS for 24 to 48 hours [19]. In our original study [19], pMSCs expressed surface markers characteristic of MSCs and differentiated into osteocytes, adipocytes and chondrocytes. To expand pMSCs, these cell preparations were cultured for 5 to 8 days in RPMI 1640 with 10% FBS, 20 ng/mL epidermal growth factor (EGF, R&D Systems, Minneapolis, MN), 20 ng/mL bFGF, 1 mM sodium pyruvate, 10 mM HEPES, 100 U penicillin/1000 U streptomycin, and 71.5 µM β-mercaptoethanol (Sigma-Aldrich). Once 75–90% confluent, the cell monolayer was enzymatically detached with 0.05% v/v trypsin-EDTA. Cells were re-plated in supplemented RPMI media described above at a density of 2500–5000 cells per cm^2^. Both bone marrow and pancreatic derived MSCs from passages 2–6 were utilized for this study. All cell cultures were maintained at 37^o^C with 5% CO_2_ in a humidified incubator.

To confirm that both bMSCs and pMSCs express the classical MSC surface antigens, cells from passages 2–6 were stained with MSC markers based on the position statement from the International Society for Cellular Therapy (ISCT) [21]. Cells were fixed in 4% w/v paraformaldehyde for 1 hour and washed with phosphate buffer saline (PBS) before primary antibody staining. Cells were stained for MSC markers CD29-PECy5 (Caltag, Carlsbad CA), CD44-FITC (Chemicon, Billerica, MA), CD73-PE, CD90-PE (BD Biosciences, Mississauga, Canada), and CD105-PE (Biolegend, San Diego, CA) as well as hematopoietic lineage markers CD11b-FITC (Abcam, San Francisco CA), CD19-PE (Abcam), CD34-FITC and CD45-PE (Caltag) for 30–60 minutes (4^o^C, protected from exposure to light). MSC marker expression was analyzed on a BD FACScalibur flow cytometer. For isotype controls, cells were also stained for IgG_1_-PE (Cedarlane, Burlington, Canada), IgG_1_-FITC (Cedarlane) and IgG_1_-PECy5 (Caltag).

### Preparation of Human Dermal Fibroblasts

Human dermal fibroblasts were prepared and expanded from normal skin samples of donors aged 35 and 60 in order to serve as a negative control cell population (Division of Plastic Surgery, University of Alberta) [22]. Fibroblasts were cultured in Dulbecco’s Modified Eagle Medium high glucose (DMEM 25 mM glucose, Gibco) and detached using 0.05% v/v trypsin-EDTA. For sub-culturing, cells were split at a ratio of 1∶6 and fibroblasts at passages 3–8 were used in this study.

### Exposure of Human Islet:MSC Co-cultures to Pro-inflammatory Cytokines

Bone marrow (bMSC) and pancreatic (pMSC) derived MSCs were enzymatically detached from culture plates, counted, and added (0.5, or 1.0×10^6^ cells) to a 100 mm low adherence culture dish (Corning) with 500 human islets in a total volume of 10 mL. Controls included islets cultured alone (± cytokines) and islets co-cultured with human dermal fibroblasts (± cytokines). The culture medium consisted of DMEM low glucose (5.6 mM glucose, Gibco) with 1% FBS, 20 ng/mL EGF, 20 ng/mL bFGF, 10 mM HEPES, 100 U penicillin/1000 U streptomycin, and 71.5 µM β-mercaptoethanol. After 24 hours, these islet:MSC co-cultures and islet:fibroblast co-cultures were exposed to a previously described cocktail of pro-inflammatory cytokines [23] including 1560 ng interferon-γ, 250 ng tumor necrosis factor-α, and 0.4625 ng interleukin 1β (specific activities 2.4×10^6^ U/mg, 5–2×10^7^ U/mg, 1.16–0.54×10^9^ U/mg respectively; Biolegend) in 10 mL volume for 48 hours.

### Characterization of Islet:MSC Co-cultures

A static incubation assay [24] was used to determine glucose responsiveness in islet controls and in co-cultures following the 48 hour cytokine exposure. Islets, islet:MSC co-cultures and islet:fibroblast co-cultures were collected and washed twice by gravity sedimentation over 30 minutes. Preparations were then divided into representative aliquots and incubated at 37^o^C for 2 hours in 1.5 mL RPMI supplemented with 2.0 mM L-glutamine, 0.5% w/v BSA and either 2.8 mM (low) or 20.0 mM (high) glucose. Tissue and medium were then separated by centrifugation and assayed for their respective insulin contents by a human insulin immunoassay (Meso Scale Discovery, Gaithersburg, MD). The insulin content of that secreted in the medium was normalized to that of the total cellular insulin content [24]. To assess total cellular insulin content, intact islets from representative aliquots were lysed then centrifuged to remove cellular debris. Cellular insulin content was measured by a human insulin immunoassay. Stimulation indices were calculated by dividing the amount of insulin released at 20.0 mM glucose by that released at 2.8 mM glucose and insulin release (% insulin content) is reported as insulin secreted at 2.8 mM or 20.0 mM glucose divided by total cellular insulin content.

### Analysis of Islet β-cell Apoptosis by Immunohistochemistry

Cytokine induced β cell damage was assessed by the co-expression of insulin and TUNEL, a marker for cell apoptosis, from human islets. After culture, paraffin sections of islets were prepared for double immunofluorescence (IF) analysis by fixation with 4% w/v paraformaldehyde (BDH Laboratory Supplies, Poole, England) and embedding in 2% w/v low melting point agarose (Sigma-Aldrich). Paraffin sections were processed and immunostained. After rehydration, antigen retrieval for tissue samples was performed in sodium citrate buffer (pH 6.0). The samples were then blocked with 20% normal goat serum (NGS, Jackson ImmunoResearch Laboratories Inc., West Grove, PA, USA) for 1 hour in the dark. For apoptosis, an APO-BrdU TUNEL Assay Kit (Invitrogen) was utilized, in which an AlexaFluor 488 labeled anti-BrdU antibody was used for detection. The same tissues were then stained with a guinea pig anti-insulin antibody (Dako, Mississauga, ON, Canada) diluted at 1/1000 in 5% NGS followed by a secondary AlexaFluor 594 mouse anti-guinea pig antibody (Molecular Probes, Eugene, OR, USA). Slides were coverslipped with Prolong Gold Anti-fade (Invitrogen) to preserve fluorescence. Negative controls included sections of the same tissues incubated without insulin primary antibodies. For the TUNEL assay, TUNEL positive and TUNEL negative control cells were stained to confirm TUNEL positivity. Slides were visualized with an Axioscope II microscope equipped with an AxioCam MRC and analyzed with Axiovision 4.6 software (Carl Zeiss, Gottingen, Germany).

### Analysis of Culture Supernatant for Cytoprotective Factors

To assess cytoprotective factors secreted by MSCs in islet:MSC co-cultures exposed to cytokines, factors were measured in the cell culture supernatant and analyzed by custom immunoassay kits (interleukin 6 (IL-6)/interleukin 10 (IL-10), hepatocyte growth factor (HGF)/vascular endothelial growth factor (VEGF), matrix metalloproteinase 2 (MMP2)/matrix metalloproteinases 9 (MMP9), Meso Scale Discovery). To determine base line levels, MSCs were cultured alone as either a cell monolayer or as cell aggregates in 6 well plates at a density of 0.2×10^6^ cells/2.0 mL. Supernatant was collected from 1 and 3 day MSC cultures, and cell debris was removed from the supernatant and collected by centrifugation (10, 000×g, 10 minutes, 4°C). Supernatant from islet:MSC co-cultures (the same used to measure insulin secreted content) was collected following exposure to cytokines and also centrifuged to remove and collect cell debris. To normalize protein content secreted into the medium to cellular DNA, cells collected from corresponding supernatant samples were assessed for DNA content. To measure DNA content, cell pellets were washed twice with citrate buffer (150 mM NaCl, 15 mM citrate, 3 mM EDTA, pH 7.4), sonicated in DNA lysis buffer (0.5% v/v Triton x-100 in pH 7.5 Tris HCl-EDTA) and aliquots of 25 µL and 50 µL were assayed in duplicate with Pico Green reagent (Molecular Probes, Carlsbad, CA) and fluorescence was detected at 485 ex./527 em. nm. [24].

### Effect of Hepatocyte Growth Factor on Human Islets Exposed to Pro-inflammatory Cytokines

Islet cultures (500 islets in 10 mL of culture medium used for islet:MSC co-cultures) were prepared as described above with recombinant human hepatocyte growth factor (10 ng/mL rhHGF, R&D Systems, Minneapolis, MN). After 24 hours, the culture media of the islet cultures was changed. The cultures were then treated with a second dose of rhHGF and exposed to pro-inflammatory cytokines [23], including 1560 ng interferon-γ, 250 ng tumor necrosis factor-α, and 0.4625 ng interleukin 1β (specific activities 2.4×10^6^ U/mg, 5–2×10^7^ U/mg, 1.16–0.54×10^9^ U/mg respectively; Biolegend) for 48 hours. Controls included islets cultured alone and islets exposed to cytokines without HGF.

### Statistical Analysis

Data are presented as mean ± SEM. Comparisons of mean values were performed with one-way ANOVA and/or Kruskal Wallis multiple comparisons tests with the level of significance set at α = 0.05. A Bonferroni or corrected Bonferroni analysis was conducted for data considered significantly different between treatment groups. All statistical analyses were performed with STATA 11 (StataCorp LP, College Station, TX).

## Results

### Characterization of Bone Marrow and Pancreatic Derived MSCs

MSC surface antigen expression [19] was analyzed by flow cytometry for passages 2–6. Both bone marrow (n = 3) and pancreatic (n = 5) derived MSCs expressed high levels of CD29, CD73, CD90 and CD105 (>90%). However, these cells did not express hematopoietic markers CD11b, CD19, CD34 and CD45 (<5%, Table 1).

**Table 1:** Characterization of cell surface antigens on human bone marrow and pancreatic derived mesenchymal stem cells.

Epitopes	bMSC (n = 3)	pMSC (n = 5)
MSC Markers		
CD29	99.5±0.2	99.6±0.2
CD44	77.4±4.9	76.7±12.5
CD73	99.8±0.0	99.9±0.1
CD90	96.4±0.6	97.4±3.0
CD105	98.4±0.5	99.5±0.3
Non MSC Markers		
CD11b	0.9±0.1	0.7±0.2
CD19	0.9±0.1	0.8±0.2
CD34	1.2±0.1	1.2±0.1
CD45	1.5±0.2	4.3±3.8

Values are expressed as mean ± SEM from MSCs between passages 2 and 6. bMSCs, bone marrow mesenchymal stem cells; pMSCs, pancreatic mesenchymal stem cells.

### MSCs Preserve Islet Function in Islet:MSC Co-cultures Exposed to Pro-inflammatory Cytokines

Glucose stimulated insulin secretion (GSIS) was used to assess human islet function in a two hour static incubation assay at 2.8 mM and 20.0 mM glucose. Islets without cytokine exposure exhibited a glucose induced stimulation index (SI) of 2.1±0.2 (n = 7) with corresponding insulin release at 2.8 mM and 20.0 mM glucose of 2.3±0.3% and 4.6±0.4% (n = 7). Cytokine exposure significantly altered GSIS. Exposure to cytokines significantly increased insulin release at 2.8 mM and 20.0 mM glucose, respectively (11.6±1.9% and 14.3±3.1%, p<0.05), resulting in a reduced SI of 1.2±0.1 (p<0.05 vs. no cytokine). In contrast, islets co-cultured with MSCs maintained GSIS, and this protective effect was also dependent on MSC dose. In particular, islets co-cultured with 1.0×10^6^ bMSCs had significantly improved GSIS compared to cytokine treated islets (SI = 2.0±0.2 and percentage insulin release at 2.8 mM and 20.0 mM glucose of 3.6±1.6% and 6.8±0.9%, n = 6, p<0.05). Similar effects were also observed with pMSCs but the effect was more robust with all doses of bMSC aggregates (Table 2). On the other hand, this protective effect was not observed when islets were co-cultured with dermal fibroblasts. Insulin release was elevated at both 2.8 mM and 20.0 mM glucose, and the stimulation index was comparable to cytokine exposed islets (Table 2). While increasing fibroblast numbers marginally decreased percentage insulin release, the values were not significantly different from cytokine treated islets. Recovery of total cellular insulin content was significantly reduced after cytokine exposure. However, recovery of insulin content from islets co-cultured with 1.0×10^6^ bMSCs was not significantly reduced compared to untreated controls (Table 2). For each co-culture experiment, independent islet samples were assessed. In addition, to determine the effect of MSCs on islets without cytokines, islets were co-cultured with 1.0×10^6^ bMSCs or 1.0×10^6^ pMSCs as independent conditions. In the absence of cytokines, GSIS from islet:MSC co-culture was not different from islet controls. In summary, the SI for islets co-cultured with bMSCs was 2.6±0.4 compared to islets alone, 2.1±0.2; and the SI for islets co-cultured with pMSCs was 2.4±0.2 compared to islets alone, 2.2±0.4.

**Table 2:** Effect of cytokine exposure on human islet total cellular insulin content and insulin secretory capacity.

	% Recovery	Insulin Release (% insulin content)		
Culture Conditions	Cellular Insulin Content	2.8 mM Glucose	20.0 mM Glucose	Stimulation Index
Islet (n = 7)	100	2.3±0.3	4.6±0.4	2.1±0.2
Islet + cytokine, (n = 7)	34.7±3.2*	11.6±1.9*	14.3±3.1*	1.2±0.1*
Islet +0.5×10^6^ bMSC + cytokine (n = 7)	52.8±5.1*	5.1±0.8†	9.0±1.4	1.8±0.2
Islet +1.0×10^6^ bMSC + cytokine (n = 6)	68.0±5.3	3.6±1.6‡	6.8±0.9‡	2.0±0.2‡
Islet, (n = 7)	100.0	2.8±0.3	5.8±0.9	2.2±0.4
Islet + cytokine (n = 7)	28.7±5.5*	14.2±1.9*	15.4±1.8*	1.1±0.1*
Islet +0.5×10^6^ pMSC + cytokine (n = 7)	35.6±7.4*	8.6±0.7§	13.6±1.5	1.6±0.2
Islet +1.0×10^6^ pMSC + cytokine (n = 6)	33.2±7.3*	6.9±0.8^||^	10.3±1.2	1.5±0.2
Islet, (n = 4)	100.0	2.0±0.4	4.3±0.4	2.3±0.3
Islet + cytokine, (n = 4)	32.2±3.9*	12.8±3.3*	16.9±5.4*	1.3±0.1*
Islet +0.5×10^6^ fibro + cytokine (n = 3)	31.7±7.7*	9.9±1.5*	12.6±0.5*	1.3±0.2*
Islet +1.0×10^6^ fibro + cytokine (n = 4)	40.3±5.9*	8.5±1.0*	13.6±2.7*	1.4±0.1*

Results are reported as % recovery of total cellular insulin content relative to untreated controls (islets alone). Islet function is assessed by a static glucose stimulated insulin release assay. The stimulation index (SI) is calculated as a ratio of insulin release at high glucose versus low glucose. Insulin release (% insulin content) is reported as insulin secreted at 2.8 mM glucose or 20.0 mM glucose divided by cellular insulin content for corresponding islets. Values are expressed as mean ± SEM.

* p<0.05 for islet vs. all conditions.

† p<0.05 for islet + cytokine vs. islet +0.5×10^6^ bMSC + cytokine.

‡ p<0.05 for islet + cytokine vs. islet +1.0×10^6^ bMSC + cytokine.

§ p<0.05 for islet + cytokine vs. islet +0.5×10^6^ pMSC + cytokine.

||p<0.05 for islet + cytokine vs. islet +1.0×10^6^ pMSC + cytokine. (bMSCs) represents bone marrow derived mesenchymal stem cells, (pMSCs) represents pancreatic derived mesenchymal stem cells and (fibro) represents dermal fibroblasts.

### Bone Marrow Derived MSCs Prevent Cytokine Induced Islet β-cell Apoptosis

**Figure 1:**
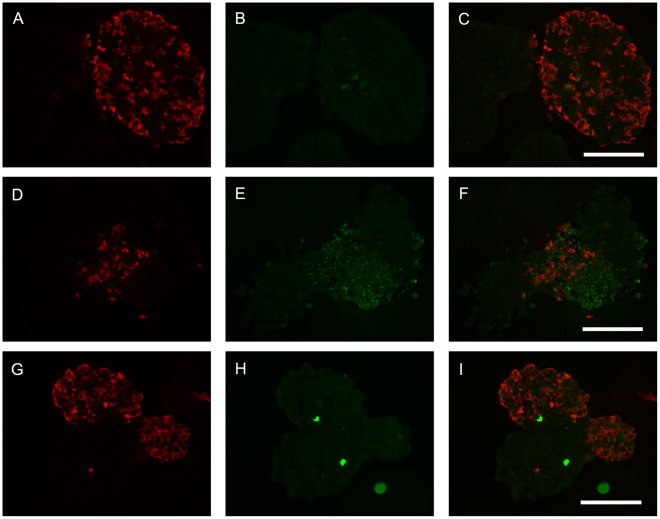
Protection of human islets from cytokine induced apoptosis by bone marrow derived MSCs. A-C) 500 Islets, D-F) 500 Islets + cytokines, G-I) 500 Islets +1.0×10^6^ bMSCs, + cytokines. Tissues were stained for insulin (A,D,G) in red and TUNEL (B,E,H) in green. The merge of the red and green images are presented in panels C, F, and I. Islets cultured without cytokines demonstrated minimal TUNEL positive cells. After cytokine exposure, the number of TUNEL positive cells increased; TUNEL and insulin co-expression was also increased with cytokine treatment. Alteration of native islet organization was observed. After cytokine exposure, co-expression of insulin and TUNEL did not increase in the islets + bMSCs group; cytokines – cocktail of IFNγ, TNFα and IL-1β described in materials and methods. Scale bar represents 100 µm.

TUNEL identifies DNA fragmentation within a cell and is an indicator of a cell undergoing apoptosis. To determine islet β cell apoptosis, tissues were co-stained for insulin and TUNEL. Islets, islets + cytokines, islets + bMSCs, and islets + bMSCs + cytokines were compared. Islets cultured without cytokines exhibited few TUNEL positive cells (Figure 1B). After pro-inflammatory cytokine exposure, insulin and TUNEL co-expression was observed in islets cultured alone (Figure 1F). The number of insulin positive cells from cytokine treated islets was also decreased compared to untreated islet controls (Figure 1D). Interestingly, other cells within the islet that did not stain for insulin were TUNEL positive. Changes to islet morphology were observed, as insulin positive cells appear less organized than untreated controls (Figure 1D, 1F). In the islets + bMSCs control, no TUNEL staining was observed (data not shown). Insulin staining and the islet morphology were similar to untreated islet controls. Cytokine exposure, however, did not induce TUNEL expression from islets co-cultured with 1.0×10^6^ bMSCs (Figure 1H, 1I). The number of insulin positive cells was comparable to control tissues, and the organization of insulin positive cells in the islet did not appear to be affected.

### Bone Marrow and Pancreatic Derived MSCs Secrete Cytoprotective Factors

Basal secretion from bMSCs and pMSCs was determined in MSC cultures without islets. MSCs secrete a variety of soluble factors that are involved in cell survival, angiogenesis and immunoregulation. Of those factors assayed, four factors (IL-6, HGF, VEGF, and MMP2) were detectable in cultures of bMSCs and pMSCs without islets or cytokines (Table 3). Control samples were the MSC culture media without MSCs. The contribution of growth factors from culture media was minimal (<0.5% of measured values) except with the IL-10 and MMP9 assays (between 1–10% of measured values). During expansion as a cell monolayer, production of HGF, VEGF and MMP-2 increased with culture time in both cell populations. On days 1 and 3, bMSC monolayers secreted higher levels of HGF (17.5±3.6 pg/ng vs. 2.2±0.7 pg/ng), VEGF (32.4±2.9 pg/ng vs. 9.4±2.5 pg/ng) and MMP2 (360.7±34.3 pg/ng vs. 114.1±9.3 pg/ng) than pMSC monolayers. Expression of IL-6 from bMSCs demonstrated increased production of IL-6 with cell expansion (13.1±1.2 pg/ng and 22.7±0.2 pg/ng), while pMSC secretion of IL-6 remained constant (16.1±6.5 pg/ng and 17.4±7.4 pg/ng) between days 1 and 3 in culture, respectively (Table 3).

**Table 3:** Basal secretion of growth factors and cytokines from human bone marrow and pancreatic derived mesenchymal stem cells.

			(pg protein/ng DNA)					
Conditions	MSC	Days Cultured	IL-6	IL-10	HGF	VEGF	MMP2	MMP9
Monolayer	b	1	13.1±1.2	0.0±0.0	2.1±0.2	12.0±2.1	114.4±6.7	0.3±0.2
Monolayer	b	3	22.7±0.2	0.1±0.0	17.5±3.6	32.3±3.9	360.7±34.3	0.5±0.2
Monolayer	p	1	16.1±6.5	0.0±0.0	0.5±0.1	2.6±0.3	81.7±2.7	0.2±0.1
Monolayer	p	3	17.4±7.4	0.0±0.0	2.2±0.7	9.4±2.5	114.1±9.3	0.0±0.0
Aggregate	b	1	0.5±0.1	0.0±0.0	1.5±0.4	1.5±0.3	47.2±6.1	0.2±0.0
Aggregate	b	3	0.3±0.1	0.0±0.0	1.5±0.4	3.4±0.8	51.7±5.7	0.2±0.1
Aggregate	p	1	9.1±3.3	0.1±0.0	3.0±0.5	2.8±0.5	112.3±4.8	0.1±0.0
Aggregate	p	3	25.6±7.3	0.1±0.0	17.9±3.2	12.6±2.6	312.0±16.2	2.3±1.1

Levels of cytoprotective factors were measured from conditioned culture media, where bone marrow and pancreatic MSCs were cultured alone as either a cell monolayer or cellular aggregates. Values obtained from cell cultures were subtracted from background levels (<0.5% of measured values for IL-6, HGF, VEGF, MMP2 and 1–10% of measured values for IL-10, MMP9) measured in culture media alone. All values are normalized to the DNA contents of each culture condition. Values are expressed as mean ± SEM (n = 3). (b) represents bone marrow and (p) represents pancreas derived MSCs.

When cultured as cell aggregates, MSCs exhibited a different secretion profile. HGF, VEGF, MMP2 and IL-6 remained detectable. However, production of these cytoprotective factors from aggregated bMSCs was reduced in comparison to monolayer bMSCs (Table 3). In addition, between days 1 and 3, expression of IL-6 (0.5±0.1 pg/ng and 0.3±0.1 pg/ng), HGF (1.5±0.4 pg/ng and 1.5±0.4 pg/ng) and MMP2 (47.2±6.1 pg/ng and 51.7±5.7 pg/ng) did not change. In contrast, pancreatic MSCs demonstrated greater HGF and MMP2 production as cell aggregates in comparison to monolayer cells. Production of IL-6 and VEGF was similar between aggregated and monolayer pMSCs at both days 1 and 3.

### Pro-inflammatory Cytokine Stimulation Increases Cytoprotective Factor Release

Levels of cytoprotective factors were also determined in islet:MSC co-cultures following pro-inflammatory cytokine exposure. IL-10 production remained low in all conditions tested. Compared to islet controls, islets exposed to cytokines had an increase in the expression of all cytoprotective factors we assessed. Islet:MSC co-cultures exposed to pro-inflammatory cytokines resulted in a further increase of these factors except MMP9 (Table 4). HGF and MMP2 levels remained low in islets with or without cytokines, while MSC aggregates alone expressed relatively high levels. In islet:MSC co-cultures, the increase in HGF and MMP2 production likely resulted from contributions by MSC aggregates. MMP9 levels were undetectable from MSC aggregates with or without cytokines. With islets, MMP9 expression was high in cytokine treated islets (25.6±6.8 pg/ng) compared to untreated islets (12.5±2.5 pg/ng). After co-culture, MMP9 levels decreased in a dose dependent manner (15.1±3.8 pg/ng, 8.5±2.4 pg/ng, Table 4). A very similar pattern was observed when human islets were co-cultured with pancreatic derived MSCs (data not shown).

**Table 4:** Secretion of growth factors and cytokines from human islet and bone marrow MSC co-cultures.

	(pg protein/ng DNA)					
	IL-6	IL-10	HGF	VEGF	MMP2	MMP9
Islet	9.6±2.7	0.0±0.0	0.1±0.0	2.8±0.5	3.4±0.5	12.5±2.5
Islet + cytokine	21.1±4.4	0.1±0.0	0.1±0.0	5.5±0.4	5.7±0.9	25.6±6.8
Islet +0.5×10^6^ bMSC + cytokine	33.8±9.5	0.1±0.0	3.2±1.0*	8.6±1.5	95.8±18.7*	15.1±3.8
Islet +1.0×10^6^ bMSC + cytokine	24.2±5.3	0.1±0.0	3.4±1.0*	7.7±0.9	94.5±19.8*	8.5±2.4
0.5×10^6^ bMSC	1.4±0.2	0.0±0.0	6.0±2.6*	6.8±0.6	144.2±21.8*	0.3±0.2*
0.5×10^6^ bMSC+ cytokine	44.6±4.8	0.2±0.0	4.4±1.0*	3.7±0.4	165.4±12.7*	0.2±0.1*

Levels of cytoprotective factors were also measured from conditioned media from islets cultured alone, with cytokines and with bone marrow MSCs with and without cytokines. Values obtained from cell cultures were subtracted from background levels (<0.5% of measured values for IL-6, HGF, VEGF, MMP2 and 1–10% of measured values for IL-10, MMP9) measured in culture media alone. All values are normalized to the DNA contents of each culture condition. Values are expressed as mean ± SEM (n = 5).

* p<0.05 for islet + cytokine vs. all conditions.

### Hepatocyte Growth Factor Preserves Islet Function After Pro-inflammatory Cytokine Exposure

When islets treated with cytokines were exposed to 2.8 mM and 20.0 mM glucose, they exhibited a loss of glucose responsiveness with a stimulation index (SI) of 1.1±0.1 (n = 5) (Table 5). Percentage insulin release at 2.8 mM and 20.0 mM glucose (10.3±1.8% and 10.9±1.5% respectively, n = 5) was also significantly elevated compared to untreated islets (2.8±1.4% and 6.0±0.8% respectively, n = 5). Addition of HGF to the culture media, however, preserved the glucose responsiveness of cytokine treated islets (SI = 1.8±0.2, p<0.05), but insulin release at 2.8 mM and 20.0 mM glucose remained elevated (8.0±1.8% and 14.1±2.6% respectively, n = 4) compared to untreated islets (Table 5). Recovery of insulin content from cytokine treated islets did not improve with the addition of HGF in culture compared to islets treated with cytokines only. The effect of HGF on islets without cytokines was also assessed. No differences in GSIS were observed as the SI for islet with HGF was 2.3±0.5 compared to the SI for islets alone, 2.4±0.4.

**Table 5:** Effect of hepatocyte growth factor (HGF) on human islet total cellular insulin content and insulin secretory capacity after exposure to pro-inflammatory cytokines.

	% Recovery	Insulin Release (% insulin content)		
Culture Conditions	Cellular Insulin Content	2.8 mM Glucose	20.0 mM Glucose	Stimulation Index
Islet (n = 5)	100	2.8±0.8	6.0±1.4	2.4±0.4
Islet + cytokine (n = 5)	37.4±6.5*	10.3±1.8*	10.9±1.5	1.1±0.1*
Islet + HGF (10 ng/mL) + cytokine (n = 4)	33.7±9.6†	8.0±1.8	14.1±2.6	1.8±0.2‡

Results are reported as % recovery of total cellular insulin relative to untreated controls (islets alone). Islet function is assessed by a static glucose stimulated insulin release assay. The stimulation index (SI) is calculated as a ratio of insulin release at high glucose versus low glucose. Insulin release (% insulin content) is reported as insulin secreted at 2.8 mM glucose or 20.0 mM glucose divided by insulin content for corresponding islets. Values are expressed as mean ± SEM.

* p<0.05 for islet vs. islet + cytokine.

† p<0.05 for islet vs. islet + HGF (10 ng/mL) + cytokine.

‡ p<0.05 for islet + cytokines vs. islet + HGF (10 ng/mL) + cytokine.

## Discussion

During islet engraftment, up to 60% of islet tissue is lost within the first 72 hours after transplantation [5], [6]. The detection of inflammation in and around the islet graft with syngeneic and autologous donors suggests that a non-specific immune response is the major factor for loss of islets [5], [6], [25]. This inflammatory response can be characterized by immune cell infiltration and elevated levels of IFN-γ, TNF-α, and IL-1β [5]–[7], [10], [26]. Because the achievement of insulin independence with the Edmonton Protocol depends on transplanting a sufficient islet mass, preserving islet mass and function can prevent early graft failure and may also decrease the requirement for multiple islet donors [3], [5]. Although chemical and pharmacologic inhibition of these cytokines can improve islet graft function [7], [10], [26], islet graft specific immunosuppression would be more desirable and could be achieved by treatment with an immunoregulatory cell such as the MSC. Described as adult connective tissue derived stem cells with the capacity to differentiate into osteocytes, adipocytes and chondrocytes, MSCs are typically expanded as a cell source for tissue replacement strategies [11]–[13]. However, the reduction in inflammation, fibrosis and cell death with MSC therapy led to the recognition that MSCs could also produce potent protective factors for tissue repair and immunomodulation [27]–[30]. Here we have proposed that the cytoprotective properties of MSCs could protect human islets from pro-inflammatory cytokines. To investigate this hypothesis, we devised a co-culture method of islets and MSCs and tested the effect on islet glucose stimulated insulin secretion (GSIS) after cytokine exposure.

Previously, the favorable effects of islet:MSC co-culture for islet function have been predominantly reported in cultures where islets and MSCs are physically separated [14], [15]. However for co-transplantation, islets and MSC are not separated in distinct compartments [31]–[33]. To better understand islet:MSC interactions for transplantation, MSCs and islets need to be cultured together in the same compartment. One approach for cell contact based co-culture is to coat islets with a layer of MSCs to provide a protective barrier to the immune response [16] or to facilitate tissue engraftment [17]. Duprez *et al*. demonstrated that MSCs could coat islets; and coating was enhanced with increased MSC numbers and time in culture [16]. In our co-culture design, we did not observe this interaction. Instead, MSCs formed aggregates, which could physically interact with islets. The formation of MSC aggregates in co-culture with human islets demonstrates that direct co-culture is a promising approach to develop islet graft specific cell therapies.

To measure the beneficial effects of this co-culture strategy, we tested islet function (GSIS) of islet:MSC aggregates exposed to pro-inflammatory cytokines. Although MSC monolayers are beneficial to islet survival and function [14], [15], several authors have reported that direct co-culture does not improve islet function [16], [17]. Here we report that aggregation of MSCs with human islets can protect human islets from pro-inflammatory cytokines, but fibroblasts are not protective for islet function. This protective effect was not restricted to bMSCs, but was also observed with pMSC. Thus, preservation of GSIS in cytokine treated human islets is MSC dependent. Examination for the percentage insulin release reveals that cytokine exposure significantly increases both basal and stimulated insulin secretion (p<0.05) compared to untreated control islets. However, the release of insulin is not glucose dependent, which suggests that increased insulin secretion may be due to the cytotoxic effects of cytokines such as disruption of cell membrane integrity (5). Hostens *et al*, also reported that cytokine exposure could increase insulin release when reported as a percentage of total cellular insulin [34]. They concluded that their cytokine treatment altered the functional state of the β cell. In contrast, co-culture of islets with MSCs prior to cytokine exposure reduces percentage insulin release and maintains glucose responsive insulin secretion (Table 2). Particularly, increasing MSC numbers to 1.0×10^6^ significantly improved islet function (p<0.05), and decreased percentage insulin secretion at 2.8 mM and 20.0 mM glucose to levels that were comparable to untreated islets. Although others have reported that direct islet:MSC interactions do not produce beneficial effects [16], [17], the difference in MSC-islet interaction in our co-culture approach (minimal islet:MSC coating) is a possible explanation for the protective effects that we observed. Nevertheless, this protection is limited because cellular insulin content was decreased in all conditions exposed to cytokines. The difference in basal insulin secretion of control and co-culture islets suggests that MSCs may protect the glucose sensing but may not affect the insulin biosynthetic function of human islets. While we have not explored the mechanisms for this loss in insulin content, the disruptive effects of IFN-γ, TNF-α, and IL-1β on insulin biosynthesis have been previously reported [5], [8], [9]. Interestingly, the recovery of cellular insulin content was greatest with bMSCs.

In addition to impairing glucose responsiveness, IFNγ, TNFα and IL-1β can be cytotoxic to β cells. As bMSCs exhibited protective effects on islet function, we assessed the ability of bMSCs to prevent β cell apoptosis in our islet:MSC co-cultures. We observed that most β cells in the cytokine treated islets group were positive for TUNEL, confirming the cytotoxicity of the pro-inflammatory cytokine cocktail. A decrease in the number of insulin positive cells was observed, correlating with our data demonstrating 34.7±3.2% cellular insulin recovery in the presence of cytokines. In contrast, when islets were co-cultured with MSCs, insulin expression was maintained and fewer TUNEL positive cells were present after cytokine treatment. Therefore we conclude that MSCs could prevent islet β cell apoptosis in the presence of cytokines. Islet structure is also important for islet function. We observed that islet morphology was altered after cytokine exposure but the morphology of islets from co-culture and controls remained intact, correlating with the reported effects on islet function. These results suggest that the mechanisms underlying the protective effect of MSCs on β cells involve the mitigation of cell death pathways and the retention of native islet morphology. Identifying the signals from islet:bMSC co-cultures may be a strategy to enhance this protection.

As MSCs but not fibroblasts from different tissue sources induce similar effects on human islet function, a common MSC specific secreted factor may mediate these effects. We investigated the cytoprotective factors IL-6, IL-10, HGF, VEGF, MMP2, and MMP9 because of their reported beneficial effects on islets [14], [15], [35] and in islet transplantation [31], [33], [36], [37]. HGF, for instance, can signal pathways regulating cell survival and insulin secretion [15]. IL-6, on the other hand, prevented the functional impairment of IFN-γ, TNF-α, and IL-1β treated mouse islets [38]. Basal secretion levels from monolayer and aggregated MSCs were initially tested. IL-6, HGF, VEGF and MMP2 were consistently detected from both pancreas and bone marrow MSC monolayers, but IL-10 and MMP9 levels were low. Expression of these factors was also greater from bMSCs than pMSCs. Berman *et al*. reported similar findings, as monkey MSCs expressed IL-6, HGF and VEGF genes; whereas IL-10 gene expression remained low during monolayer expansion [37]. The three dimensional microenvironment of aggregated MSCs is unique from the two dimensional MSC monolayer. We wanted to determine whether basal production of soluble factors was markedly different with aggregated MSCs. In general, aggregated MSCs also express IL-6, HGF, VEGF and MMP2. An intriguing trend emerged in which bMSC aggregates had markedly decreased cytoprotective factor expression but pMSCs exhibited increased expression of these same factors (Table 3). This difference in cytoprotective factor production suggests that MSC function is dependent on tissue source and culture method as a cell monolayer or cellular aggregates. To better define the factors mediating protection of islets, we examined the production of these cytoprotective factors from islet:MSC co-cultures. As bMSCs exhibited the greatest protective effect, we selected the islet:bMSC co-cultures to determine expression profiles. HGF and MMP2 were MSC specific, and production increased with increasing numbers of MSCs (Table 4). We also observed that while MMP9 levels decreased, islet function improved with increasing MSC numbers, which suggests that bMSCs may protect by mitigating excessive MMP9 expression. Others reported that islets cultured in medium composed of IL-6, TGF-β, HGF and VEGF had significantly improved function [15]. In our co-culture system HGF, MMP2 and MMP9 appeared to be important MSC dependent factors associated with improved islet function after cytokine treatment. Addition of HGF to cytokine treated islets resulted in preservation of glucose responsiveness based on the stimulation index (p<0.05). Basal and stimulated insulin release, on the other hand, was elevated compared to untreated islets (p<0.05). In addition, recovery of insulin content was not improved with HGF (Table 5); whereas, islets co-cultured with bMSCs demonstrated better insulin content recovery than cytokine treated islets (Table 2). As HGF alone was unable to completely reproduce the effects of bMSC, we did not proceed to inhibit the activity of HGF in co-culture. Based on these results, we believe that other factors in addition to HGF such as MMP2, MMP9 and TGF-β may be important for cytoprotection of islet function.

In summary, MSC aggregates preserved GSIS and prevented islet β cell apoptosis of pro-inflammatory cytokine treated islets. The absence of this protective phenomenon with human dermal fibroblasts strongly suggests that preservation of GSIS is MSC dependent. Assessment of the secreted factors from MSCs demonstrates that HGF, MMP2 and MMP9 are possible secreted factors mediating protection. However, addition of rhHGF to islet cultures reveals that HGF alone cannot replace the beneficial effects of MSCs. Although the mechanism of protection is unclear, future studies can address the ability of MSCs to attenuate inflammation mediated β-cell graft dysfunction. Replicating these results in vivo will help develop a strategy to administer MSCs for clinical islet transplantation and to prolong graft survival and function.
